# Application of Antibiotics/Antimicrobial Agents on Dental Caries

**DOI:** 10.1155/2020/5658212

**Published:** 2020-01-31

**Authors:** Wei Qiu, Yujie Zhou, Zixin Li, Tu Huang, Yuhan Xiao, Lei Cheng, Xian Peng, Lixin Zhang, Biao Ren

**Affiliations:** ^1^Department of Stomatology, Nanfang Hospital, Southern Medical University, Guangzhou 510515, China; ^2^State Key Laboratory of Oral Disease, West China Hospital of Stomatology, National Clinical Research Center for Oral Diseases, Sichuan University, Chengdu 610064, China; ^3^Department of Operative Dentistry and Endodontics, West China Hospital of Stomatology, Sichuan University, Chengdu 610041, China; ^4^West China School of Stomatology, Sichuan University, Chengdu 610041, China; ^5^Chinese Academy of Sciences Key Laboratory of Pathogenic Microbiology and Immunology, Institute of Microbiology, Chinese Academy of Sciences, Beijing 100101, China; ^6^State Key Laboratory of Bioreactor Engineering, East China University of Science & Technology, Shanghai 200237, China

## Abstract

Dental caries is the most common oral disease. The bacteriological aetiology of dental caries promotes the use of antibiotics or antimicrobial agents to prevent this type of oral infectious disease. Antibiotics have been developed for more than 80 years since Fleming discovered penicillin in 1928, and systemic antibiotics have been used to treat dental caries for a long time. However, new types of antimicrobial agents have been developed to fight against dental caries. The purpose of this review is to focus on the application of systemic antibiotics and other antimicrobial agents with respect to their clinical use to date, including the history of their development, and their side effects, uses, structure types, and molecular mechanisms to promote a better understanding of the importance of microbial interactions in dental plaque and combinational treatments.

## 1. Introduction

Dental caries, or “tooth decay,” is the most prevalent chronic infectious disease in the oral cavity [1]. Dental caries is the predominant cause of tooth loss in children and young adults and is also the primary cause of tooth root breakdown in the elderly. According to a statistical data analysis by the World Health Organization (WHO), the prevalence of dental caries is 60–80% in children and almost 100% in adult population [2]. The oral cavity forms a unique ecological niche for micro-organisms, most of which accumulate on dental surfaces to form dental plaque (oral biofilm). Cariogenic bacteria that can ferment carbohydrates to produce acid and further demineralize the tooth surfaces are the primary aetiologic agents of dental caries [3–5]. *Streptococcus mutans*, *lactobacilli*, *Actinomyces* spp. and some other anaerobic bacteria are considered to be the primary cariogenic agents involved in the development of dental caries [6]. Ecologic shifts, including the increase of these pathogenic florae in dental plaques, result in faster demineralization than remineralization [7]. Dental caries not only affects oral health, but also correlates with some other system diseases, such as diabetes, indicating that the prevention and treatment of dental caries are important to mitigate this global health risk [8].

## 2. History of Dental Caries

Dental caries is an ancient disease in humans that can be traced back to 12000–3000 years BC (before Christ) according to archaeological findings [9]. A record from 5000 BC described a “tooth worm” as the cause of caries in India, Egypt, Japan, and China [10]. In ancient China, people developed many traditional methods for caries prevention. For instance, they used arsenic trioxide to relieve tooth pain, which was used until modern society [11]. In the 16th century, Antonie van Leeuwenhoek suggested that micro-organisms were involved in dental caries when he first saw the bacteria in his own plaque under a microscope [12]. In the 19th century, Miller proposed that micro-organisms in the oral cavity can utilize carbonhydrates that lead to acid production and promote the demineralization of teeth [13]. This chemical parasitic aetiology promoted the bacteriological study of dental caries. In 1924, Clarke isolated streptococci from human carious lesions and named them *S. mutans*, further claiming that this type of bacterium is involved in the development of dental caries [14]. By 1960, Keyes confirmed the involvement of this specific bacterium in dental caries using a hamster model. With the further investigation of the aetiology of dental caries, three factors, which describe food (fermentable carbohydrates), host (a susceptible tooth surface), and caries-causing bacteria, were proposed by Keyes [15]. In 1976, Newbrun revealed that time was also an important factor that plays a significant role in caries aetiology, which together formed the modern four factors of dental caries aetiology [16].

Bacteria (dental plaque) are considered to be the primary factor among the four caries aetiologic factors. The primary evidence in support of this view can be traced back to the results of a number of classic experiments, such as (1) bacteria isolated from the oral cavity can demineralize the enamel and dentin in vitro; (2) in a hamster model, extensive caries can develop in erupted molars, while unerupted molars remain caries-free until they were exposed to the oral microbiota; and (3) germ-free rats failed to develop caries even when maintained on a cariogenic diet, while control animals developed extensive decay when fed the same diet. In light of these data, the use of antibiotics or antimicrobial agents is an effective strategy for the prevention and treatment of dental caries [17–21].

## 3. Systemic Antibiotics

Antibiotic treatment began in the mid-twentieth century in the form of sulfa-containing drugs and drugs derived from microbial natural products, such as penicillin, which was discovered in 1941. Subsequently, antibiotics have been used to meet the challenges posed by bacterial infections in clinical and pharmacological research [22–25]. Early in the prevention or treatment of dental caries, systemic antibiotics showed potential efficacy [26, 27]. We have highlighted some systemic antibiotics, including penicillin, tetracyclines, metronidazole, macrolides, and clindamycin, describing their application, mechanisms, side effects, and resistance.

### 3.1. Penicillin

Penicillin, the earliest discovered and most widely used *β*-lactam antibiotic, is derived from the *Penicillium* mould and can inhibit the synthesis of the peptidoglycan layer of the bacterial cell walls by irreversibly binding to the active sites of penicillin-binding proteins (PBPs) [28]. Penicillin is effective against strains of the gram-positive *Streptococci* and *Staphylococci*, as well as some gram-negative bacteria [29, 30]. The first use of penicillin to treat dental caries dated from 1946, when McClure and Hewitt reported that penicillin inhibited caries in rats [31]. Four years later, Zander reported that penicillin showed caries inhibition in children [32]. In the 1980s, penicillin G or penicillin V was the first choice of antibiotics for the treatment of dental infections of typical aetiology [33]. However, the use of penicillin can cause some side effects, such as diarrhoea, hypersensitivity, nausea, rash, neurotoxicity, and urticaria [34]. Another major problem is the resistance of bacteria to *β*-lactam antibiotics. Bacteria can produce a new PBP gene named mecA that encodes PBP2a, the function of which is similar to that of other PBPs, but it has low bounding affinity to *β*-lactams [35].

### 3.2. Tetracyclines

Tetracyclines are a group of broad-spectrum antibiotics with the ability to inhibit protein synthesis by binding to the 30S ribosomal subunit in the mRNA translation complex [36, 37]. In 1945, chlortetracycline became the first tetracycline to be identified. However, tetracycline appears to become incorporated into human teeth, causing discoloration [38, 39]. Tetracycline staining was first reported in the mid l950s, less than a decade after the introduction and widespread use of the antibiotics [40]. In 1963, the United States Food and Drug Administration issued a warning regarding the use of such antibiotics for pregnant women and young children since teeth are most susceptible to tetracycline discoloration during their formation [41, 42]. The side effects of tetracycline include cramps or burning of the stomach, diarrhoea, sore mouth or tongue, skin photosensitivity, headache rarely, and vision problems, with damage to the kidneys also having been reported [43].

### 3.3. Metronidazole

Metronidazole, a nitroimidazole class antibiotic and an antiprotozoal medication that, is used either alone or with other antibiotics to treat pelvic inflammatory disease, oral infections, endocarditis, etc. [44]. Metronidazole can inhibit nucleic acid synthesis when it is reduced by disrupting DNA [45]. The reduction of metronidazole often occurs in anaerobic bacteria, and metronidazole is more effective against anaerobic organisms such as *Fusobacterium*, *Bacteroides*, *Clostridium*, *Prevotella*, and *Peptostreptococcus* species [46, 47]. Metronidazole is available as a cream for the mouth and has a wide spectrum of bactericidal action against oral obligate anaerobes, even against isolates from infected necrotic pulps [48, 49]. More than 99% of the bacteria present in carious lesions and infected root dentin were not recovered in the presence of metronidazole in in vitro experiments [50, 51]. The first commercial use of metronidazole occurred in 1960 in France. Side effects of metronidazole, including nausea, a metallic taste, headaches, flushing of the skin, tachycardia, loss of appetite, and shortness of breath, have been reported [52].

### 3.4. Macrolides

Macrolides, a polyketide class of natural products that consist of a large macrocyclic lactone ring, are typically used to treat infections caused by *β*-haemolytic *streptococci*, *pneumococci*, *staphylococci*, and *enterococci*, having a slightly wider antimicrobial spectrum than penicillin [53, 54]. Macrolides prevent peptidyl transferase from adding the growing peptide attached to tRNA to the next amino acid and can inhibit ribosomal translation by reversibly binding to the P site on the 50S subunit of the bacterial ribosome [55]. Side effects include myopathy, long QT syndrome, enterohepatic recycling, and cholestasis [54]. Erythromycin is a macrolide antibiotic for the treatment of a number of bacterial infections. Keyes showed that caries-active dams become caries-inactive when treated with erythromycin and erythromycin treatment can decrease the amount of plaque formed by 35% after one week [56].

### 3.5. Clindamycin

Clindamycin, a semisynthetic derivative of lincomycin that, is primarily used to treat anaerobic infections caused by susceptible anaerobic bacteria, including dental, respiratory tract, skin, soft tissue, and peritonitis infections [57]. Clindamycin was first synthesized in 1966 and began being marked in 1968. Clindamycin is an excellent alternative for patients who are allergic to penicillin or for penicillin-resistant infections, particularly if resistant anaerobes are suspected. Clindamycin functions as an inhibitor of bacterial protein synthesis by disrupting ribosomal translocation [58]. It has been reported that susceptibility-guided antibiotics with benzylpenicillin plus clindamycin and successive mitral annuloplasty can result in the inhibition of *S. mutans* and *Lactobacillus acidophilus* growth in vitro [59]. Side effects include diarrhoea, pseudomembranous colitis, nausea, vomiting, abdominal pain or cramps, and contact dermatitis [60].

## 4. Other Typical Antimicrobial Agents

Many systemic antibiotics were not developed to treat oral bacteria or are not specific to treat oral diseases. The application of systemic antibiotics has gradually reduced during recent decades, with other antimicrobial agents having been developed to target oral bacteria that cause oral diseases, such as fluoride, chlorhexidine, quaternary ammonium salts, and antimicrobial peptides (AMPs).

### 4.1. Fluoride

Fluoride is the simplest anion of fluorine but is one of the most successful cavity prevention agents especially for preventing dental caries [61]. Fluoride is typically supplemented in small quantities to drinking water or, products such as mouthwashes, toothpastes, and oral supplements [62]. The anticaries actions of fluoride remain controversial. A popular mechanism is that fluoride ions contact the mineral of the tooth surface and increase remineralization to prevent the acid-induced demineralization caused by cariogenic bacteria [24]. Fluoride has also been indicated to inhibit enolase, a key enzyme in the glycolytic pathway. Inhibition of enolase results in the growth inhibition and reduced acid production of oral streptococci, such as *S. mutans* [63, 64]. However, dental and skeletal fluorosis, as well as the development of fluoride-resistant oral bacteria, has led to a reconsideration of the administration of fluoride [65].

### 4.2. Chlorhexidine

Chlorhexidine is, a cationic polybiguanide that was one of the first antiseptic agents proposed for dental caries and has proved to be the most effective [66]. Jacinto et al. investigated the plaque-inhibiting activity of chlorhexidine and definitively showed that initial dental caries did not develop when students rinsed with a solution of sucrose 9 times a day over a 22-day period while simultaneously rinsing with a 0.2% chlorhexidine solution twice daily in 1972 [67]. To date, chlorhexidine remains the “gold standard” of antiplaque agents. Chlorhexidine is active against gram-positive and gram-negative bacteria, facultative anaerobes, aerobes, and yeasts by damaging the inner cytoplasmic membrane [66, 68]. With regards to the inhibition of plaque, chlorhexidine can block the acidic groups of glycoproteins present in saliva to reduce plaque adhesion. Chlorhexidine can reduce the binding of bacteria to tooth surfaces adsorbing chlorhexidine to extracellular polysaccharides or competing with calcium ions agglutination in plaque [69]. However, chlorhexidine causes genotoxicity by inducing DNA damage in leukocytes, kidney cells and oral mucosal cells, and it can also induce the apoptotic cellular apoptosis [70, 71].

### 4.3. Quaternary Ammonium Salts

Quaternary ammonium salts are widely used as antimicrobial agents, surfactants, fabric softeners, and antistatic agents [72]. In the 1970s, quaternary ammonium salts were first administered to inhibit oral plaque by being incorporated into mouth rinses [73]. Quaternary ammonium salts are typically used as additives in dental materials to give them antimicrobial abilities [74, 75]. The antimicrobial mechanism of quaternary ammonium salts is not fully understood. A common explanation is that the positive charge of quaternary amines bind to the negatively charged bacteria cells to disturb the electric balance and can also promote the bacterial lysis by binding to bacterial membranes [76–78]. Side effects of quaternary ammonium salts administration include gastrointestinal symptoms, coma, convulsions, hypotension, and death [79].

### 4.4. Antimicrobial Peptides (AMPs)

Antimicrobial peptides (AMPs) are short, cationic host-defence molecules that exert potent antimicrobial activities against a broad spectrum of microorganisms. In the oral cavity, there are many natural AMP molecules, such as hBD-1,2,3 (human *β*-defensin-1,2,3), SMAP (sheep myeloid antibacterial peptide), LL-37 (a cathelicidin), nisin and histatins, which possess antimicrobial activities against oral pathogenic bacteria and biofilms [80]. However, many artificially designed AMPs have been developed to control caries progression and pulpal infections because of the high cytotoxicity and poor tissue distribution of natural AMPs [81]. The antimicrobial mechanism of natural AMPs is cell permeabilization followed by membrane disruption, which depends on their relatively strong electrostatic attraction to negatively charged bacterial cells [82].

### 4.5. Remineralizing Agents

Dental caries is a cyclic event with periods of demineralizations and remineralization, where remineralization process is a natural repair mechanism of teeth to restore the presence of minerals to the hydroxyapatite (HAP) crystal lattice in ionic forms [83]. At present, numerous types of remineralizing agents have been researched and many are being used clinically to treat dental caries, with significantly predictable positive results. In addition to fluorides mentioned above, remineralizing agents have been broadly classified into the following categories: calcium phosphate materials (such as alpha tricalcium phosphate and sodium calcium phosphosilicate), nanoparticles (such as nanoHAP particles, ACP nanoparticles, and nanobioactive glass materials), polydopamine, oligopeptides, theobromine, and arginine [84].

## 5. Combinational Therapy and Its Future

Four factors associated with acidogenic theory for the causation of dental caries indicate that dental caries is a multifactor infectious disease. To realize the full-potential preventive or treatment approaches towards dental caries, the combinational strategy can lead to new caries managements. Below, we discuss the use of combinations of antimicrobial and remineralizing agents and the application of probiotics which can reduce the tooth decay.

### 5.1. Antimicrobial Agents with Remineralizing Agents

The first ICNARA conference (International Conference on Novel Anticaries and Remineralizing Agents) held in Chile in January 2008 highlighted two key components of caries management: antibacterial agents and remineralizing agents [80]. After four years, the second ICNARA conference concluded that antibacterial agents are necessary, since remineralization alone was insufficient to deal with the caries challenge especially in high-risk individuals [85, 86]. Fluoride is widely used in clinics to promote enamel remineralization. Fluoride acts as a glycolytic enzyme inhibitor to reduce enamel destruction caused by acid [87], while also acting as a transmembrane proton carrier. Both of these mechanisms inhibit bacterial induced cytoplasmic acidification [87]. Fluoride is a widely recognized dual functional anticaries agent, acting on both tooth hard tissue and oral microbes [88]. Apart from this, dental caries is closely associated with the microbial metabolism of carbohydrates, allowing caries development to be inhibited by antimicrobial drugs as previously mentioned [89].

In addition to fluoride, nanoparticles of silver (NAg) and amorphous calcium phosphate (NACP) can also reduce acid production in dental plaque and enhance remineralization [90]. NAg can inhibit the growth of bacteria via the perturbation of cell membranes and through the toxicity of Ag to some cellular enzymes, whereas NACP can release calcium/phosphate ions to promote remineralization. These ions can remarkably increase the NACP filler level in adhesives [91–95]. To increase the antibacterial activities of Nag and NACP, other antibacterial materials, such as quaternary ammonium methacrylates (QAMs) and chlorhexidine (CHX), have been incorporated. For example, the addition of NAg and quaternary ammonium dimethacrylate (QADM) into bonding agents during the restoration of tooth cavity has been shown to effectively inhibit the reproduction of cariogenic bacteria, especially *S. mutans*, and reduce the viability, metabolic activity, and the acid production of oral microorganisms [96]. NACP combined with QADM can also inhibit bacteria growth, reduce the amount of biofilm matrix, and decrease acid production [97]. Moreover, some cells on NACP-QADM nanocomposites have been shown to disintegrate [98]. In addition to QAMs, it has been shown that the antimicrobial activity of ACP (amorphous calcium phosphate) and CaF2 nanocomposites can be greatly increased when added to CHX fillers, while the ability of biofilm formation was significantly reduced [98–100].

These combinatorial treatments against two or more cariogenic factors described above provide a new strategy for dental caries prevention and treatment.

### 5.2. Probiotics and Reducing Cariogenic Bacteria

Antibiotics and antimicrobial agents cannot kill all of the offending pathogenic bacteria and can even wipe out many other oral flora, which may lead to negative consequences, such as the overgrowth of antibiotic-resistant bacteria or fungal pathogens. An ideal approach to caries treatment would be one that could selectively inhibit cariogenic pathogens while leaving the oral ecosystem intact. For example, scientists have developed a selective targeting molecule that only attaches to the organism of interest, such as *S. mutans*, or other selected pathogens [101]. Then, an antibacterial molecule is optimized and chained to the targeting molecule. The combined unit then selectively removes the target pathogens, such as *S. mutans*, preventing the recurrence of dental caries [102, 103].

In addition, the success of probiotics in controlling gastrointestinal diseases has led to the use of probiotics to control the growth of cariogenic bacteria in the oral cavity. The concept of probiotics is that an adequate amount of specific bacteria can promote host health [85]. Currently, the known mechanisms of probiotic activity can include the following: (1). competing for binding sites on the tooth surface; (2) competing for nutrients; and (3) producing antimicrobial compounds to inhibit other oral bacteria, such as hydrogen peroxide, bacteriocins, and adhesion inhibitors [104, 105]. For example, the administration of *Lactobacillus rhamuosus* GG strain to milk was shown to reduce initial caries in kindergarten children in Helsinki, Finland [106]. *L. reuteri* ATCC 55739 and *Bifidobacterium* DN-173 010 showed significant growth inhibitory effects against cariogenic *S. mutans* in saliva [107]. Baca-Castanon et al. [108] identified several new strains of *Lactobacillus* with good antimicrobial activity against *S. mutans* and good binding characteristics to oral mucin. The antagonistic effects among various oral streptococci may also be a probiotic approach to shift the ecology of the oral cavity [109–111]. For example, *S. salivarius* strains can produce bacteriocin-like inhibitory substances with a broad spectrum of activity against cariogenic streptococci including *S. mutans*. *S. oligofermentans* is a bacterium that appears to be inversely correlated with the presence of *S. mutans* within dental plaque samples and was shown to produce hydrogen peroxide with lactic acid as the substrate, leading to the inhibition of *S. mutans* growth [112, 113].

Researchers have also developed a replacement therapy by constructing functional mutated strains of *S. mutans* through gene engineering and DNA recombination technology and then replacing the wild-type isolate of *S. mutans* in the oral cavity to prevent dental caries [114]. Among these mutants, the non-acid-producing *S. mutans* mutants that lack the ability to metabolize fermentable carbohydrates to produce organic acids have been well developed. For example, *S. mutans* strain BCS3-L1 cannot produce acid due to deletion of lactic acid dehydrogenase, significantly reducing its the cariogenic abilities compared with the parent strain, and it can even produce an antibiotic called mutacin 1140 that acts against other *S. mutans* strains in the oral cavity [115–118]. Thurnheer et al. deleted the glucosyltransferase-C- (GTF-C-) encoding gene of *S. mutans* to reduce the production of extracellular polysaccharides (EPS), which decreased significantly a mixed biofilm [119].

The study of probiotics is a novel area of study in oral medicine that aims to significantly reduce the levels of oral pathogenic microorganisms. Unfortunately, the probiotics studied to date have not permanently colonized the oral cavity [120, 121]. A highly promising way to utilize probiotics will be the use of a combination of antibiotics or antimicrobial agents with probiotics to prevent or treat dental caries based on the concept that broad spectrum antibiotics or antimicrobial agents wipe out the native oral flora, after which the probiotics therapy can promote the rebuilding of a healthy oral ecology [122].

### 5.3. Consideration of Microbial Interactions in Dental Plaque

The human oral cavity is in a state of coexistence with a microbial community [123]. Varieties of interactions between microbes normally maintain a balance in a healthy environment, while the overgrowth of conditional pathogens along with their increased virulence factors in the oral microenvironment disrupts this balance, leading to infectious diseases in the oral cavity, such as dental caries [113, 124–126]. The effect of microbial diversity and the interactions in microbial communities cannot be ignored. For example, with respect to the interaction between *Candida albicans* and oral streptococci, the overuse of broad antibiotics or antibacterial agents occasionally causes the overgrowth of fungi, such as *C. albicans*, in the oral cavity [127–129]. *C. albicans* is also found in dental carries lesions among children and the elderly and plays an important role in the development of dental carries [130–134]. Broad spectrum antibiotics or antibacterial agents cannot kill *C. albicans*. Furthermore, *C. albicans* can even increase the cariogenic virulence of oral bacteria, such as *S. mutans* [119, 135–139]. In addition to the synergistic interactions between *C. albicans* and oral streptococci, multi-species biofilms can promote antibiotic and antifungal resistance [140, 141]. However, clinical antibiotics consistently function against fungi or bacteria despite the cross-kingdom interactions. In addition, viruses may play a vital role in shaping microbial populations, but this phenomenon has been poorly studied in oral environments. The optimal antibiotics should be the ones that can shape the dental plaque to alter the pathogenic plaque into a healthy one [131, 134, 142, 143].

### 5.4. Antibiotics Resistance

Dental caries is a significant public health problem in many parts of the world, and at present, the first choice for the prevention of caries and periodontal diseases remains the mechanical removal of oral biofilms. At the same time, the use of antibiotics has offered a new means for doctors to overcome dental caries [144]. However, the use of antibiotics alone cannot completely inhibit the demineralization and may cause subsequent infection due to the resistance of several types of bacteria to drugs [145]. The formation of microbial biofilms, such as dental plaque, is an important reason for the failure of antimicrobial therapy and the promotion of antibiotic resistance [146, 147]. However, the molecular mechanisms underlying the survival of biofilm cells are still not completely understood. There are three potential reasons for this issue: the first is biofilm-specific protection against oxidative stress; the second concerns the biofilm-specific expression of efflux pumps to pump out antibiotics; and the third is protection provided by matrix polysaccharides that can reduce the diffusion of antibiotics, which may play a significant role in antibiotic resistance [148, 149]. These mechanisms are associated with both bacterial and fungal biofilms and are often surprisingly similar between distantly related organisms [150].

**Figure 1:**
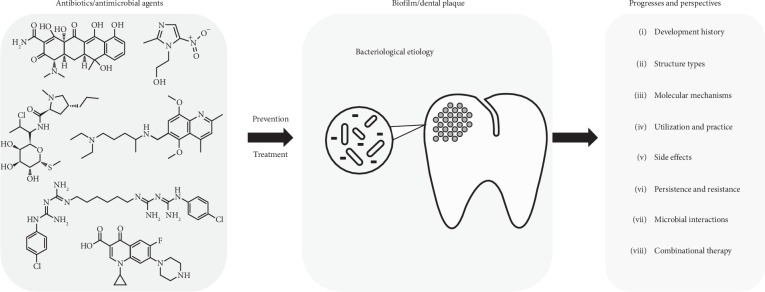
Antibiotics and antimicrobial agents are increasingly being developed to fight against dental caries. In this review, Qiu et al. focused on discussing the application of systemic antibiotics and other antimicrobial agents with their clinical use to date, including the history of their development and their side effects, uses, structure types, and molecular mechanisms to promote a better understanding of the importance of microbial interactions in dental plaque and combinational treatments.

Apart from these mechanisms, recent studies have shown that fluoride-resistant microorganisms, which are relatively different from fluoride-sensitive ones in growth and metabolic activity, may be another key factor in antibiotic resistance [65]. The fluoride-resistant microorganisms can be detected in xerostomia patients who have been treated with a much higher concentration of fluoride [151]. Therefore, the existence of fluoride-resistant microorganisms, especially fluoride-resistant *S. mutans*, will influence the effects of caries prevention and treatment and even lead to the failure of caries management [65]. Furthermore, the common treatment (antibiotics or antimicrobial agent treatment) cannot completely inhibit the growth and metabolic activity of bacteria. Oral microorganisms left in cavities not only can induce the recurrence of dental caries but also do harm the pulp system, particularly when cariogenic bacteria, such as *S. mutans*, are the dominant microorganisms in dental plaque [152]. Even though cavities are filled completely, the marginal leakage may promote secondary dental caries [153]. In consideration of these factors, there may be promising for finding an effective method to reduce the persistence of cariogenic bacteria (Figure 1).

## 6. Conclusion and Future Perspectives

Dental caries is the most common oral infectious disease through early childhood to old age [154]. Compared with systemic antibiotics, the use of drugs that target the specific cariogenic microorganism is a relatively ideal therapy for dental caries. Furthermore, a new concept is that the human oral cavity is in a state of coexistence with a microbial community. However, few studies have investigated the effects of antibiotics on the oral microbial community and their relationships with oral disease. Based on the solid relationships between microbiome and diseases, the maintenance of the ecological balance is key to the treatment of oral diseases and is also a future direction in the development of new antimicrobial agents used in the oral cavity. In this case, the use of probiotics has excellent potential to reshape the oral microbial community.

Another concern in this area is that the overuse of antibiotics can cause resistance or persistence [155]. To avoid the development antibiotic resistance or persistence, the use of a combination of two or more antibacterial agents (even antifungal agents), especially those with different mechanisms, is a practical and fast means of developing new therapies for dental caries.
